# The Antibiofilm Role of Biotics Family in Vaginal Fungal Infections

**DOI:** 10.3389/fmicb.2022.787119

**Published:** 2022-05-26

**Authors:** Angela Boahen, Leslie Thian Lung Than, Yi-Linn Loke, Shu Yih Chew

**Affiliations:** Department of Medical Microbiology, Faculty of Medicine and Health Sciences, Universiti Putra Malaysia, Serdang, Malaysia

**Keywords:** *Candida* biofilms, probiotics, postbiotics, prebiotics, synbiotics, synergistic therapy, vaginal dysbiosis, vulvovaginal candidiasis

## Abstract

“Unity in strength” is a notion that can be exploited to characterize biofilms as they bestow microbes with protection to live freely, escalate their virulence, confer high resistance to therapeutic agents, and provide active grounds for the production of biofilms after dispersal. Naturally, fungal biofilms are inherently resistant to many conventional antifungals, possibly owing to virulence factors as their ammunitions that persistently express amid planktonic transition to matured biofilm state. These ammunitions include the ability to form polymicrobial biofilms, emergence of persister cells post-antifungal treatment and acquisition of resistance genes. One of the major disorders affecting vaginal health is vulvovaginal candidiasis (VVC) and its reoccurrence is termed recurrent VVC (RVVC). It is caused by the *Candida* species which include *Candida albicans* and *Candida glabrata*. The aforementioned *Candida* species, notably *C. albicans* is a biofilm producing pathogen and habitually forms part of the vaginal microbiota of healthy women. Latest research has implicated the role of fungal biofilms in VVC, particularly in the setting of treatment failure and RVVC. Consequently, a plethora of studies have advocated the utilization of probiotics in addressing these infections. Specifically, the excreted or released compounds of probiotics which are also known as postbiotics are being actively researched with vast potential to be used as therapeutic options for the treatment and prevention of VVC and RVVC. These potential sources of postbiotics are harnessed due to their proven antifungal and antibiofilm. Hence, this review discusses the role of *Candida* biofilm formation in VVC and RVVC. In addition, we discuss the application of pro-, pre-, post-, and synbiotics either individually or in combined regimen to counteract the abovementioned problems. A clear understanding of the role of biofilms in VVC and RVVC will provide proper footing for further research in devising novel remedies for prevention and treatment of vaginal fungal infections.

## Introduction

A plethora of studies attribute the presence of a *Lactobacillus* dominated vaginal microflora as the hallmark of health in the female reproductive tract. Aside this, an imbalance or absence of these species lead to various disorders as observed in vaginal dysbiosis infections. Vaginal dysbiosis is characterized by the imbalance of the microbial communities in the vagina, which results in the invasion of pathogens, leading to infections (Zhou et al., 2009; Datcu, 2014; van de Wijgert and Jespers, 2017). This condition has been associated with pelvic inflammatory diseases, increased risk for acquisition and transmission of sexually transmitted infections (e.g., cervicitis, HIV), pre-term birth and complications (Fredricks et al., 2007; Roberts et al., 2011a; Fettweis et al., 2019). The common symptoms of vaginal dysbiosis include abnormal vaginal discharges, pruritus, redness of the vulva, increased pH, fishy vaginal odor and vaginal burns after sex and urination (Zhou et al., 2009; Itapary Dos Santos et al., 2019; Ma et al., 2019; Yano et al., 2019). Generally, most vaginal infections are highly recurrent but localized (Paladine and Desai, 2018). However, in rare cases, these infections can spread from the vagina to adjacent regions like the cervix, as observed in cervicitis (Keshavarz et al., 2001; Marrazzo et al., 2006). Although cervicitis is sexually acquired, and an inflammatory condition of the cervix that is commonly linked with *Chlamydia trachomatis* or *Neisseria gonorrhoeae*, there have been reports of these organisms not detected in infected women. Surprisingly, there have been reports and evidence of bacterial vaginosis (BV) being strongly associated with cervicitis. The presence of this vaginal infection increased the risk of acquiring an upper vaginal tract infection by a threefold as reported (Keshavarz et al., 2001; Marrazzo et al., 2006). BV is the most reported form of vaginal dysbiosis and occurs when the normal *Lactobacillus* species in the vagina are replaced with anaerobic bacteria, resulting in reduced levels of hydrogen peroxide (H_2_O_2_) and other organic acids usually present in the vagina (Syed and Braverman, 2004; Koumans et al., 2007; Haahr et al., 2016; van Teijlingen et al., 2020). Aside BV, other prevalent forms of vaginal dysbiosis include aerobic vaginitis (AV), trichomonas vaginitis (TV) and vulvovaginal candidiasis (VVC; Landers et al., 2004; Workowski et al., 2015; Rowley et al., 2019). AV is distinguished by the abundant presence of aerobic, enteric bacteria but short of lactic acid bacteria (LAB) in the vaginal microflora. These commensal aerobic bacteria (e.g., *S. agalactiae*, *Escherichia coli*, *Staphylococcus aureus*, *Enterococcus faecalis*, *Klebsiella pneumoniae*, *Gardnerella vaginalis*, and *Atopobium vaginae*) are known to be of intestinal origin (Donders et al., 2002; Haahr et al., 2016; Ma et al., 2022). This is a possibility since the anus is closest to the vagina. Therefore, in the course of poor personal hygiene, there is the likelihood for gut microbes to travel into the vagina. According to the study by Donders and Rezebarga (2011), AV has been observed in 5%–24% of reported vaginal infections and 8%–11% in pregnant women (Donders and Rezebarga, 2011). TV on the other hand is a non-viral sexually transmitted infection also dependent on vaginal ecology such as absence or decrease in H_2_O_2_-producing lactobacilli and presence of BV (Sutton et al., 2007; Baeten et al., 2009; Rathod et al., 2011; Kissinger, 2015). In a study by Moodley et al. (2002), authors describe the disturbance of the vaginal ecology, initiated by a TV infection to also be responsible for the change in normal vaginal flora and may therefore, make one susceptible to BV and HIV infections (Moodley et al., 2002). The abovementioned vaginal infections are segregated into vaginosis and vaginitis. Vaginosis (e.g., BV) does not cause inflammatory signs in infected women and on the other hand, vaginitis (e.g., VVC, AV, and TV) causes inflammation in the vagina of infected women.

In this review, the relevant keywords used for literature search included: *Candida* species, biofilms, vaginal dysbiosis, vaginitis, VVC, RVVC, antifungal drug resistance, role of -biotics in vaginal health, synergistic treatment for the abovementioned problem statement. The search engines, mainly Google Scholar, PubMed, Scopus and ScienceDirect were used to search and retrieve available published literatures, related to antibiofilm role of biotics family in vaginal fungal infections. There was no limitation on the publication dates due to the scarcity of data on role of -biotics in VVC and RVVC. Written articles in English, which include original research articles, case reports and chapters in books were included in this review. However, unpublished data and materials that are not in English were excluded.

### Vulvovaginal Candidiasis

Vulvovaginal candidiasis is the second most reported form of vaginal dysbiosis and affects 70%–75% of healthy women of reproductive age (Sobel, 2017). It is generally characterized by inflammation of the vagina, increased pH, whitish dense vaginal discharge, itchiness coupled with soreness of the vagina, and severely hampers the quality of life of infected women (Apalata et al., 2014; Yano et al., 2019). Unfortunately, 9% of women with VVC experience recurrence of VVC (RVVC), defined as four or more repeated episodes of the infection within a year (Foxman et al., 2013). Vulvovaginal candidiasis is an incident of vaginal dysbiosis caused by the overgrowth of *Candida* species, including *Candida albicans* and non*-albicans Candida* species (NACS) like *Candida glabrata*, *Candida krusei*, *Candida tropicalis*, and *Candida parapsilosis* (Landers et al., 2004; Sobel, 2016; Babu et al., 2017). Under normal circumstances, the ubiquitous *Candida* species are opportunistic fungal pathogens found as part of the normal vaginal microflora and poses no threat to the host. Yet, unfavorable conditions such as pH fluctuation, immunosuppression, poor personal hygiene can cause changes in the growth of the normal bacterial flora, leading to fungal overgrowth and invasiveness (Beigi et al., 2004; Gulati and Nobile, 2016; Silva et al., 2017). Hence, vaginal dysbiosis. VVC is significantly prevalent as symptomatic in immunocompromised women (e.g., diabetic, pregnant, and HIV patients) with vaginal infections (Ray et al., 2007; Apalata et al., 2014; Abdul-Aziz et al., 2019). In pregnant women, VVC tends to manifest and become symptomatic because of the fluctuation in the host immune system, in response to increased production of glycogen and elevated estrogen levels. This may lead to an increased risk of pregnancy complications, such as premature labor and birth (Meizoso et al., 2008; Roberts et al., 2011b; Mølgaard-Nielsen et al., 2016). Vulvovaginal candidiasis can be classified as either uncomplicated or complicated. Uncomplicated VVC can be described as the vaginal fungal infection which is susceptible to commercially available antifungals and antimycotic treatments (Sekhavat et al., 2011; Qin et al., 2018). This form of VVC is most often at times associated with *C. albicans*. However, the complicated VVC is recurrent with four or more repeated episodes of the infection. Complicated VVC, is severe and almost untreatable with antimycotic treatment. Although *C. albicans* are mostly associated with complicated VVC, NAC such as *C. glabrata* are also isolated in patients with complicated VVC. Complicated VVC is linked with immunocompromised individuals such as acquired immunodeficiency syndrome (AIDS) patients, diabetic and pregnant women (Spinillo et al., 1997; Malazy et al., 2007; Ray et al., 2011; Waikhom et al., 2020).

Despite the variety of antifungal agents available for VVC, there are actually very few that compare their efficacy along with the risk of developing *Candida* resistance or RVVC (Lírio et al., 2019). It is believed that RVVC is potentially caused by either *Candida* reinfection or a vaginal relapse (Rosati et al., 2020; Willems et al., 2020). In comparison to vaginal reinfection, vaginal relapse is the more well-accepted theory in explaining the event of RVVC. The emergence of persister cells within the *Candida* species, especially NACS such as *C. glabrata* following antifungal treatment makes the complete eradication of the pathogen extremely difficult. As a result, this gives room for a possible reinfection. Nevertheless, *Candida* species also develop adaptive characteristics that allow them to overcome antifungal assaults. For instance, *Candida* species are able to form biofilms that protect them from unfavorable environments while allowing them to adhere and persist on both biotic and abiotic surfaces (Finkel and Mitchell, 2011; Fanning and Mitchell, 2012). The inability of antifungals to penetrate these biofilms and expose the fungal cells within would inevitably lead to development of antifungal resistance.

## Role of *Candida* Biofilms in VVC

Microbial biofilms have significant impacts on many human diseases and over the years, have become one of the most researched areas in hope of discovering countermeasures for this problematic issue. This is mainly because, biofilms provide a sanctuary for opportunistic pathogens to thrive and exhibit their virulence. Biofilms are defined as aggregates of microorganisms in which complex communities of microbial cells are frequently embedded in a self-produced matrix of extracellular polymeric substances (EPS; Vert et al., 2012). Fungal biofilms are a cluster of yeast cells surrounded by an extracellular matrix (ECM), which provides structural support for attachment between cells, different surfaces, and their environment (Chandra et al., 2001; Ramage et al., 2001; Al-Fattani and Douglas, 2006; Mitchell et al., 2016). Initiation of *Candida* species biofilm formation involves an initial attachment to a surface, morphogenesis/filamentation, cell to cell interactions to build cell densities, biofilm maturation and dispersal to seed on new sites. Migration of yeast cells to new sites enables them to not only establish dominance and increase their fungal burden, but also help them to escape localized hostility like in the case of biofilm disruption. Biofilm formation is a trait of *Candida* species but however, strain dependent. For example, *C. albicans* matured biofilms are composed of blastopores, pseudohyphae, hyphae whiles *C. glabrata* biofilms are made up of a cluster of only yeast cells (Chandra et al., 2001; Ramage et al., 2002; Silva et al., 2009). Alas, biofilm formation has been proposed as one of the most important virulence factors of *Candida* species in causing infections generally (Mayer et al., 2013; Tsui et al., 2016; Cavalheiro and Teixeira, 2018). Clinically, biofilm infections can be extremely difficult to eradicate due to their resistance to antimicrobial agents, host defenses and also persistence on mucosal surfaces. Much recently, fungal biofilms have been implicated in VVC, particularly in the uncommon cases of treatment failure and recurrence (Harriott et al., 2010; Wu et al., 2020). As *Candida* species are part of the normal vagina flora in women, it is safe to say that they only become symptomatic when there is an increase in fungal burden due to physiological factors that favor them. With the increase of fungal burden, the devious *Candida* species especially *C. albicans*, build up their cell’s densities and display virulent characteristics like hyphal formation and biofilm formation. The transition from planktonic state to hyphal and matured biofilms states by *Candida* species, is a mechanism in causing infections. This adaptation mechanism by fungal species involves the secretion of molecules that facilitates their cell-to-cell interactions such as signal molecules and quorum sensing molecules (QSMs; Wongsuk et al., 2016; Barriuso et al., 2018; Padder et al., 2018). For instance, *Candida* species are known to secrete QSMs such as farnesol, tyrosol, phenylethanol, and tryptophol (Kruppa, 2009; Wongsuk et al., 2016). These diverse QSMs are produced during the biofilm formation process to govern cell development and morphology in biofilm communities. In actuality, quorum sensing involves the production and release of low molecular weight signaling compounds called autoinducers. When cell density threshold is reached, autoinducers initiate a collective and coordinated expression of genes for the development of biofilms which are influenced by many environmental factors like temperature, pH, cell density, nutrients composition and concentration (Sudbery, 2011; Elias and Banin, 2012; Padder et al., 2018). These autoinducers, also known as QSMs, are released during quorum sensing to regulate microbial activities like morphogenesis, pathogenesis, competence, biofilm formation and development (Albuquerque and Casadevall, 2012; Polke et al., 2015; Padder et al., 2018). In spite of the fact that quorum sensing was exclusively associated with bacterial species, the realization of the role of farnesol on filamentous growth of *C. albicans*, threw light on the essence of quorum sensing and QSMs in fungal species. This is due to the fact that when amassed, farnesol can prevent filamentous growth and biofilm formation of *C. albicans*. This accumulation occurs *via* passive diffusion across the membrane, involving efflux pumps and specific transporters (Hogan, 2006; Sprague and Winans, 2006; Riekhof and Nickerson, 2017). So far, it is known that farnesol is secreted by *Candida* species such as *C. albicans* and *C. dubliniensis* (Weber and Ruhnke, 2010) and produced as a secondary product of sterol biosynthesis (Hornby et al., 2001). According to Polke et al. (2018), farnesol also influences the expression of genes involved in cell wall maintenance, response to oxidative stress and phagocytosis, antifungal resistance, and heat shock (Polke et al., 2018). Aside this, other QSMs such as tyrosol accelerates hyphal development and germ-tube formation in *C. albicans* (Nickerson and Hornby, 2006).

It is worth noting that, a vast number of studies conducted on biofilm-associated vaginal infections, have been investigated *in vitro* and only a small number of studies have demonstrated biofilms growth on the vaginal epithelium *in vivo* (Harriott et al., 2010; Gao et al., 2016; Yan Y. et al., 2019; Wu et al., 2020). However, recent studies have shifted the focus to more *in vivo* and *ex vivo* models of fungal biofilm-associated vaginal infections. Based on a few studies conducted to identify the role of *Candida* biofilms in VVC, results have proven the ability of *C. albicans* specially, to form biofilms on vaginal epithelium of murine animal models, both *in vivo* and *ex vivo* (Harriott et al., 2010). These observations were characterized by the increase in fungal burden, presence of ECM, hyphae formation and dense biofilm architectures (Harriott et al., 2010; Yan Y. et al., 2019; Wu et al., 2020). Distinctly, Harriott et al. (2010) was the first study to provide evidence that *C. albicans* can form biofilms on vaginal epithelium, other than on abiotic surfaces. In their study, scanning electron microscopic examination displayed hyphae of *C. albicans* on vaginal tissue of immunocompromised BALB/C mice. Microscopic analysis demonstrated typical biofilm architecture with a mixture of yeast and hyphae forms of *C. albicans*, surrounded by ECM (Harriott et al., 2010). In other *in vivo* studies, the increase in fungal burden of *C. albicans* resulting in adherence, hyphae formation and overexpression of biofilm promoting genes like hyphae wall protein (*HWP*) and agglutinin-like sequence (*ALS*) have also been reported (Gao et al., 2016; Yan Y. et al. (2019). In addition, the study conducted by Wu et al. (2020), also exhibited the formation of *C. albicans* (i.e., reference and clinical strains) biofilms *in vivo* and *ex vivo*. In their *in vivo* studies, both the reference and the clinical isolates of *C. albicans* formed biofilms on the vaginal epithelium of BALB/c female mice used in their study. This was 72 h post-inoculation with *C. albicans* fungal cell suspensions. Histological assessment of the dissected vagina showed the development of numerous microabscesses with fungal and neutrophils infiltrations, which had penetrated the lumen and mucosa of the vaginal tissues. There was also formation of endocytosed hyphae in the mucosa which was visualized by periodic acid-Schiff stain. This further induced local inflammatory responses in the infected mice by the increase in inflammatory effectors (alarmin S100A8 and interleukin-1β). Aside this, the biofilm-defective mutants strains of *C. albicans* used in the study, did not form biofilms on the vaginal epithelium of the mice, and vaginal tissues of mice were comparable to uninfected mice vaginal tissues (Wu et al., 2020).

**Figure 1:**
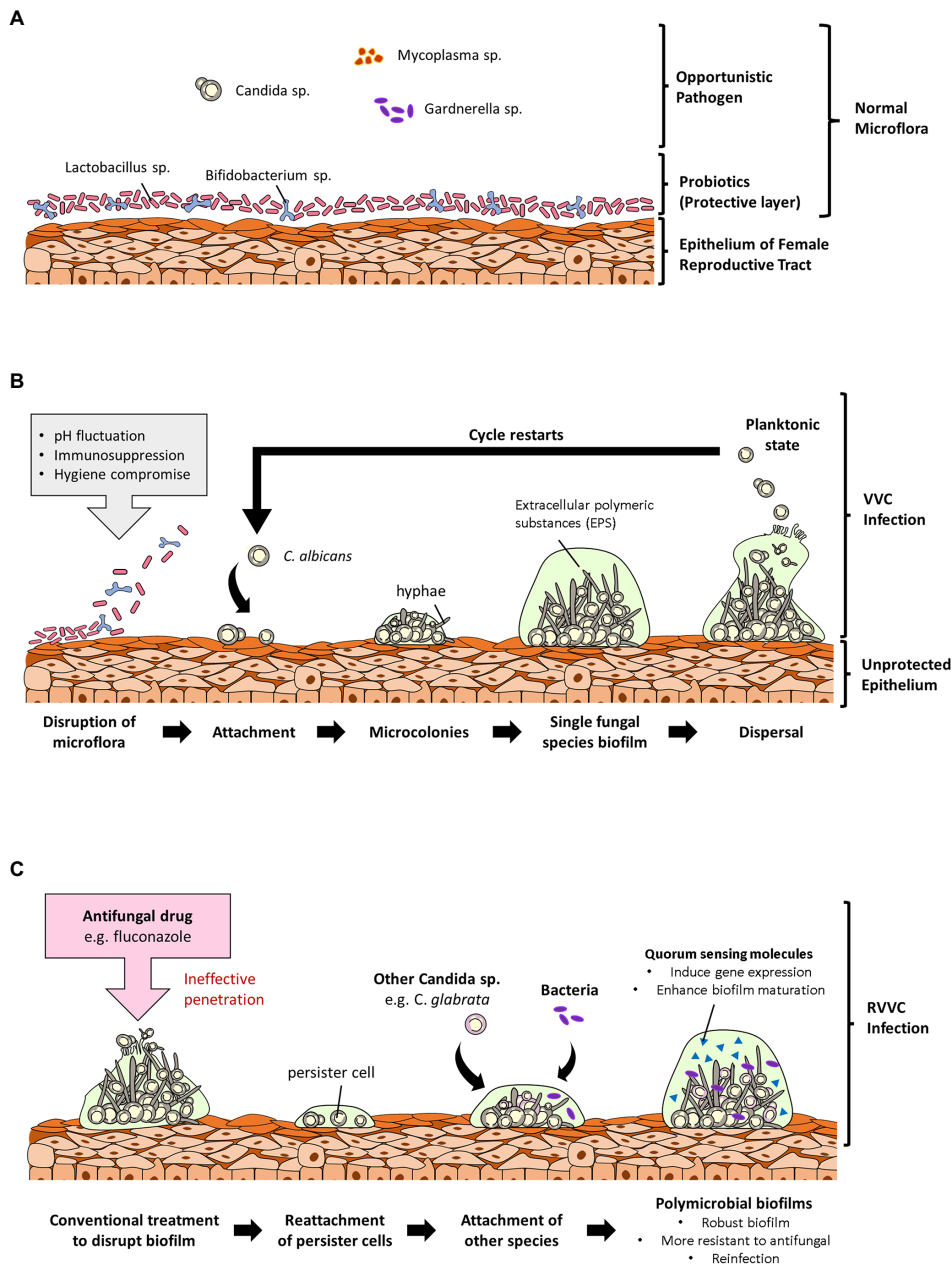
Overview of the role of fungal biofilms in vulvovaginal candidiasis (VVC). **(A)** Shows a normal vaginal microflora with microorganisms existing cordially. In the normal vaginal microflora, probiotic strains are the primary colonizers, and they confer protection to the vagina by forming a protective layer over the vaginal epithelium. **(B)** Shows an incidence of vaginal dysbiosis due to unfavorable conditions such as pH fluctuations, immunosuppression, and poor personal hygiene. Opportunistic *Candida* species then adheres to the vaginal epithelium due to the disruption of the protective layer and forms biofilms leading to VVC. **(C)** Describes the ineffectuality of commercial antifungals to completely disrupt biofilms leaving behind persister cells. Persister cells of different *Candida* species can attach to each other or to pathogenic bacteria and form polymicrobial biofilms resulting in a reinfection and RVVC. These polymicrobial biofilms are denser and more difficult for antifungals to penetrate to targeted pathogens.

With regards to biofilm formation of NACS such as *C. glabrata* on vaginal epithelium, there have been studies of *C. glabrata* isolates, attaching and forming biofilms on human vaginal epithelial cells (VK2/E6E7). These studies mainly proved the ability of *C. glabrata* reference and clinical strains, to be able to form biofilms on vaginal mucosa surfaces (Cavalheiro et al., 2018, 2021). Considering adhesion to be a crucial and an initial step of *Candida* species biofilm formation, it was reported in the study by Cavalheiro et al. (2018), that, the epithelial adhesin 3 (*EPA3*) gene was overexpressed in *C. glabrata* clinical isolate used in the study. In *C. glabrata*, the EPA family is mainly responsible for its adhesion on biotic and abiotic surfaces (De Las Peñas et al., 2003). Due to this, there was decrease in the intracellular accumulation of azoles (i.e., voriconazole and fluconazole; Cavalheiro et al., 2018). More *in vivo* studies are however encouraged to prove the formation of fungal biofilms on the vaginal epithelium. This is because, there is a possible association of vaginal fungal biofilms in persistent virulence in the host and resistance to antifungals due to their perseverance on mucosal surfaces. Hence, understanding the development, morphology and physiology of *Candida* biofilms have great medical significance. Physicians who are well informed about the relevance of biofilms in vaginal infections can use this knowledge to make decisions that will influence treatment outcomes. A representative diagram of fungal biofilms role in VVC and its reoccurrence is illustrated below in Figure 1.

## Current Treatment Options for VVC

Vulvovaginal candidiasis is commonly treated with over-the-counter (OTC) antifungals since they are potent against acute or uncomplicated VVC (Mendling et al., 2015). These OTC agents are mainly topical agents like vaginal tablets, creams, suppositories, pessaries, and oral drugs. Antifungal agents are generally fungistatic (inhibiting) and fungicidal (killing) to the growth of pathogenic yeast. Fungi possess sterols (e.g., ergosterol) that are organic compounds responsible for mediating their cellular and physiological functions, thereby, a target for antifungal agents (Onyewu et al., 2003; Borelli et al., 2008; Dupont et al., 2012). Basically, azoles exhibit fungistatic activities against fungal species by lowering ergosterol levels in their cell walls, *via* the inhibition of enzymes that enhance ergosterol biosynthesis. Azoles also elevate levels of reactive oxygen species. These actions by azoles in turn inhibit growth and development of fungal species (Kobayashi et al., 2002; Delattin et al., 2014; Tits et al., 2021). However, some studies indicate that some azoles such as fluconazole (FLZ) can exhibit dose- dependent fungicidal activity on *C. albicans* (Lee and Lee, 2018). Unlike azoles, polyenes (e.g., nystatin, amphotericin B) and echinocandins (e.g., caspofungin, micafungin, and anidulafungin) can be fungistatic and fungicidal. They function by attaching to the ergosterol in the cell wall and causing leakage through pore formation (Matsuoka and Murata, 2003; Gray et al., 2012; Efimova et al., 2014). This subsequently results in cell death. In particular, echinocandins inhibit β-(1,3)-D-glucan synthesis in fungal cell walls which results in osmotic cell death (Morris and Villmann, 2006). These OTC antifungals are usually administered orally or locally and show good short-term efficacy. The oral route of administration has been the preferred choice for most women since its more convenient to administer as compared to intravaginal route. Besides, FLZ is the most preferred and common azole for *Candida* infections treatments (Pfaller et al., 2010). Antifungal susceptibility testing has further indicated that FLZ is effective against various *Candida* species such as *C. albicans*, *C. parapsilosis*, and *C. tropicalis* (Pfaller and Diekema, 2007). Unfortunately, it is now well known of the reduced effectiveness of antifungal agents and other antimicrobial agents which has become a major public health threat. Therefore, novel treatments for VVC and RVVC are being widely investigated. The recommended intravaginal and oral treatments for VVC patients are summarized in the Table 1 below.

**Table 1:** Recommended intravaginal and oral agents for VVC treatment.

**Over-the-counter intravaginal agents**	**Description**
Butoconazole	2% cream 5 g intravaginally in a single application
Butoconazole	2% cream 5 g intravaginally for 3 days
Clotrimazole	1% cream 5 g intravaginal for 7–14 days
Clotrimazole	2% cream 5 g intravaginal for 3 days
Clotrimazole	100 mg vaginal tablet for 7 days
Clotrimazole	100 mg two vaginal tablets for 3 days
Miconazole	2% cream 5 g intravaginally daily for 7 days
Miconazole	4% cream 5 g intravaginally daily for 3 days
Miconazole	100 mg vaginal suppository one suppository daily for 7 days
Miconazole	200 mg vaginal suppository one suppository for 3 days
Miconazole	1,200 mg vaginal suppository one suppository for 1 day
Nystatin	100,000-unit vaginal tablet, one tablet for 14 days
Tioconazole	6.5% ointment 5 g intravaginally in a single application
Terconazole	0.8% cream 5 g intravaginally daily for 3 days
Terconazole	80 mg vaginal suppository one suppository daily for 3 days
**Oral agent**	**Description**
Fluconazole	150 mg orally in a single dose

Source: Division of STD Prevention, National Centre for HIV/AIDS, Viral Hepatitis, STD, and TB Prevention, Centres for Disease Control and Prevention.

### Antifungal Resistance of *Candida* Species in VVC

Primarily, frequent usage of antifungals can eventually lead to the ineffectuality of drugs against pathogens especially when biofilms are involved. The failure of antifungals to completely eradicate biofilms leads to the survival of persister cells and this can result in reinfections. It has been shown that persister cells are capable of enhanced biofilms development and express higher virulence (Uppuluri et al., 2010). Also, the overuse of antifungals leads to antimicrobial resistance and factors that promote overuse include self-diagnosing and self-medicating by infected women due to unrestricted access to these OTC drugs (Ferris et al., 2002; Bartlett et al., 2013). Moreover, research also proclaims the formation of fungal biofilms empower the cells within to withstand high dosage of antifungals and also resist host immune defenses especially in immunocompromised individuals (Andes et al., 2004; Schinabeck et al., 2004; Broaders et al., 2013). Regrettably, *C. albicans* developing resistance to azoles in infections including vaginitis have been reported (Marchaim et al., 2012; Adjapong et al., 2017; Sherry et al., 2017; Whaley et al., 2017; Jin et al., 2018; Singh et al., 2019). In the early 2000s, Perea et al. (2001) and Vazquez et al. (2001) in their various studies recorded failure of antifungals against *Candida* species, specifically *C. albicans* in immunocompromised patients subjected to long term use of FLZ before other NACS such as *C. glabrata*, *C. parapsilosis*, *C. dubliniensis*, *C. tropicalis* were also vindicated (Perea et al., 2001; Vazquez et al., 2001). In the clinical study by Marchaim et al. (2012), vaginitis cases caused by *C. albicans* from the year 2000 to 2010 were enrolled to determine resistance to antifungals and reoccurrence of the infection. Response to antifungals was determined by broth microdilution. Results showed 25 women with FLZ resistant vaginitis and recurrence with 16 of 25 women exposed to low-dose weekly FLZ maintenance therapy. All patients were clinically controlled successfully, even though the treatment was difficult and often prolonged. Although FLZ resistant *C. albicans* was previously considered rare, the study recorded 25 cases over an 11-year period, indicating it was an emerging problem (Marchaim et al., 2012). In the year 2015, another clinical study by Nasrollahi et al. (2015), enrolled cases of 52l vaginal samples obtained from females infected with *C. albicans* vaginitis. The azole susceptibility test was determined by disk diffusion method and inhibition zone for FLZ according to the Clinical Laboratory for Standards Institute protocols. Polymerase chain reaction was used to evaluate FLZ resistance in *C. albicans* and expression of cell wall mannoprotein (*PIR1*) gene, a structural protein of the cell walls of *C. albicans*. Results showed that out of the 52 isolates, 49 (94%) were resistant to FLZ. Overexpression of *PIR1* gene was detected in 47 (96%) FLZ resistant *C. albicans* isolates (Nasrollahi et al., 2015). In the study by Adjapong et al. (2017), *Candida* species isolated from RVVC patients were shown to be insensitive to FLZ as compared to *Candida* isolates from VVC patients. Adjapong et al. (2017), reported a decreased susceptibility of *C. albicans* isolates and NACS isolates (i.e., *C. parapsilosis* and *C. tropicalis*) in RVVC patients as compared to VVC patients, with *C. glabrata* maintaining resistance in both infections (Adjapong et al., 2017). In 2021, a clinical study by Arastehfar et al. (2021), was conducted within a 1-year duration. The aim of the was to determine the frequency of RVVC among women referred to a gynecology hospital in Tehran, and the association of azoles, specifically clotrimazole and FLZ treatments, with the emergence of FLZ resistant *Candida* isolates. The study observed that the widespread use of OTC azoles can influence FLZ therapeutic success. Results also indicated that about 53% of the patients experienced RVVC. *C. albicans* and *C*. *glabrata* constituted approximately 90% of the yeast isolates (72 patients) and *C. albicans* constituted 81.2% of FLZ resistance isolated from the women (Arastehfar et al., 2021). Contrastingly, *C. glabrata* may be inherently resistant to all azoles and about 20% of the strains are known to develop resistance during therapy and prophylaxis with FLZ (Pfaller and Diekema, 2007; Pfaller et al., 2015). *Candida auris*, the new superbug has been shown to express 93% resistance to FLZ (Lockhart et al., 2017). Recently, *C. auris* has been isolated from a vaginitis case involving a 26-year-old Indian woman. In that case report, *Candida* species from the vaginal swab was identified as *C. auris* by sequencing of the fungal internal transcribed spacer region. Furthermore, antifungal susceptibility testing revealed that, the *C. auris* isolate was resistant to FLZ, amphotericin B and clotrimazole. The isolate also exhibited strong biofilm forming ability which further contributed to its multidrug resistance (Krishnasamy et al., 2021). *Candida auris* is an emerging superbug, often misidentified as other yeasts. It is distinguished by its multidrug resistance to a variety of antifungals and is often associated with nosocomial infections. However, recent clinical reports have isolated *C. auris* from patients’ urine, stool, vagina, vaginal fluid, and rectum (Welsh et al., 2017; Forsberg et al., 2019).

To our knowledge, no antifungals have been reported to be able to fully eradicate *Candida* biofilms in VVC. Biofilms are resilient and extremely difficult to be completely eliminated by the current antifungal arsenal. In fact, the antifungal resistance to azoles (i.e., fluconazole, itraconazole, voriconazole, posaconazole) is particularly conspicuous, while echinocandins (i.e., caspofungin, micafungin, and anidulafungin) and liposomal amphotericin B are relatively more effective. Although many *in vitro* studies have showed that echinocandins and liposomal amphotericin B have potent antibiofilm activity against *Candida* species, their roles in full eradication of *Candida* biofilms *in vivo* still remain uncertain (Kuhn et al., 2002; Schinabeck et al., 2004; Soustre et al., 2004; Cocuaud et al., 2005; Cateau et al., 2008; Hoyer et al., 2018).

### Mechanism of Resistance to Antifungals by *Candida* Species

The resistance to antifungals, especially azoles by *C. albicans* may result from modifications of target enzymes due to mutations in the lanosterol 14-alpha-demethylase (*ERG11*), the gene that encodes lanosterol demethylase, which is the target of the azoles (Flowers et al., 2015). Other techniques may involve the active efflux pump of the drug out of the cell *via* the activation of transport proteins (Sanglard et al., 1997; White et al., 2002; Saini et al., 2005; Rawal et al., 2013). Undoubtedly, the reduced access of antifungals to the targeted pathogens due to fungal biofilm formation, reduces the drug’s potential. This is described as a drug exclusion technique by the pathogens in the biofilm matrix. Also, phenotypic changes resulting from nutrient limitation/low growth rate, or surface-induced gene expression and formation of polymicrobial biofilms by *Candida* species (Jabra-Rizk et al., 2004, Cavalheiro and Teixiera, 2018). Since this review discusses the role biofilms play in VVC and RVVC, the formation of polymicrobial biofilms as a resistance mechanism by fungal species will be delved into further.

Generally, microbes are often found in mixed-species communities as polymicrobial biofilms and interactivities are usually mutualistic, communalistic, or antagonistic (Hall-Stoodley et al., 2004). Clinically, polymicrobial infections involving different species such as bacteria and fungi, are unyielding to treatment and as such, can lead to an increase in mortality rates as compared to single biofilm species (Harriott and Noverr, 2011; Peters et al., 2012; McKloud et al., 2021). Fungal–bacterial interactions to form mixed species biofilms is dependent on the microbial environ and can be beneficial or antagonistic to the species involved. For example, in the study by Adam et al., 2002 the presence of *C. albicans* enhanced the growth of *Staphylococcus epidermidis in vitro*. In turn, *S. epidermidis* inhibited penetration of FLZ into the mixed species biofilm through ECM production (Adam et al., 2002). Furthermore, mixed species biofilm interaction can influence specific proteins and genes expression, which could help explain induction of antimicrobial resistance and virulence (Peters et al., 2010). Additionally, a clinical study which was carried out within a 3-year period (January 2001 to December 2003) with 127 participants to determine formation of biofilms on intrauterine devices (IUDs) showed that, most of the microbes recovered from the cultures originated from the vagina. These included aerobic and anaerobic bacteria including *C. albicans*. This might mean polymicrobial biofilm formation on these devices can contribute to recurrence of genital infections and drug resistance (Pál et al., 2005; Harriott and Noverr, 2011). In the course of mixed species biofilm formation, QSMs play a major role by directing the synthesis of active compounds and interactions between microorganisms. This includes behavioral response towards each other, physical interaction between microbial cells such as provision of hyphae by *C. albicans* as attachment sites for bacteria cells in mixed species biofilms and physiological alterations of the local environment like change in oxygen content and pH (Atriwal et al., 2021; Pohl, 2022). Polymicrobial biofilms involving *C. albicans* and NACS generally also show inherent antifungal resistance and are more difficult to eliminate. For instance, polymicrobial biofilms consisting of *C. glabrata* and *C. albicans* has a more complex biofilm structures and enhanced antifungal resistance (Olson et al., 2018). The interplay between *C. glabrata* and *C. albicans* is also mutualistic. *C. albicans* serves as a platform upon which *C. glabrata* attaches and in turn, *C. albicans* benefits from the ability of *C. glabrata* to attach to numerous surfaces due to production of adhesins (Tati et al., 2016; Timmermans et al., 2018). Without doubt, the ability of opportunistic *Candida* species to stick to each other to form an alliance is an obvious advantage to compete with the primary colonizers, that is, probiotic strains (*Lactobacillus* and *Bifidobacterium* species) that confer a protective layer on the vaginal epithelium. In addition, NACS such as *C. tropicalis* and *C. dubliniensis* can attach to *C. albicans* to benefit from dual-species biofilm growth *in vitro* (Pathirana et al., 2019). Rossoni et al. (2015) also provided evidence of mixed biofilms of *C. albicans*, *C. glabrata*, and *C. krusei in vitro* and *Galleria mellonella* larvae (Rossoni et al., 2015). Sherry et al. (2017) also demonstrated that *Candida* species isolated from RVVC patients have the ability to form heterogeneous biofilms and exhibit increased resistance to FLZ (Sherry et al., 2017) *in vitro*. In the study, a total of 300 fungal isolates from vaginal swabs were cultured to form biofilms. The antifungal susceptibility testing was afterwards carried out to determine the minimum inhibitory concentration of FLZ against the isolated *Candida* species. Their results proved that vaginal fungal isolates are all able to form biofilms regardless of species. *C. albicans* was the most dominant species isolated (71%) in their study, a higher number of the *C. albicans* isolates displayed reduced susceptibility to FLZ in RVVC patients due to the formation of a polymicrobial biofilm with the other *Candida* isolates. In short, the presence of a mixed fungal biofilm involved in RVVC aids in the development of resistance to FLZ (Gao et al., 2016; Sherry et al., 2017).

Following biofilm formation, that is, single and mixed species, been established as a major mechanism of *Candida* species in causing infections and developing resistance in vaginal infections, throwing light on biofilm prevention and disruption can be a leap to solving antifungal drug resistance in VVC/RVVC. Also, synergistic therapy to suppress biofilm infections by combining high doses of antifungals with regimen that weaken or disrupt biofilms, is encouraged in this review. These synergistic agents will penetrate the site of biofilm infections to get to the pathogens within, since dispersed cells are unchallenging to eliminate. Furthermore, administering of anti-quorum sensing molecules that can cripple of social activities amongst fungal species will in the end control and prevent biofilm formation. Role of polymicrobial biofilms in vaginal reinfections is illustrated in Figure 1.

## Role of Biotics in Preventing Biofilms Prevalence in Vulvovaginal Candidiasis

### Probiotics

The high tolerance of vaginal *Candida* biofilms to commercially existing antifungals has ignited interest in possible alternatives such as the employment of -biotics, plant extracts and nanoparticles. These approaches are to provide an improved regimen for the treatment of VVC and RVVC (Ludwig et al., 2018; Souza et al., 2018; De Gregorio et al., 2019; Liao et al., 2019; Liu et al., 2020; Parolin et al., 2021). In this review, we will discuss the role of the Biotics family, that is; probiotics, postbiotics, prebiotics and synbiotics play major roles in the war against vaginal yeast biofilms. Over the years, probiotics have played significant roles in improving human health and are recognized as safe (O’Toole et al., 2017). To date, the microorganisms which have been isolated and usually used as probiotics are LAB, which are native members of a healthy human microbiota (Fijan, 2014). A profusion of studies has reported application of probiotics as antibiofilm agents, adjuvants, vaccines, and other therapeutic options to stimulate the host’s immune response to pathogens (Berg and Mertz, 2010; Bassaganya-Riera et al., 2012; Tsilingiri et al., 2012; Wang et al., 2012, 2014; Chew et al., 2015). The human vagina is inhabited by a wide range of microbes with majority being lactobacilli species, particularly in healthy women. However, the vagina microbiota can change composition rapidly especially during vaginal dysbiosis. This leads to infections and cohabitation or dominance of microorganisms with pathogenic potential like in fungal infections. Recurrent infection may also be due to the elimination of the commensal organisms in the vagina by the antifungal, thereby increasing dominance of already existing pathogens (Kalia et al., 2020). This is one of the main reasons for considering the use of probiotics, to restore and replenish the normal bacterial flora, to lower the risk of reinfection. The ability of lactobacilli strains to adhere to vagina mucosa is an advantage in temporarily populating the vagina and creating an environment conducive to the restoration of the host’s indigenous lactobacilli rather than a return of pathogens.

Probiotics are live microorganisms that when administered in adequate amounts, confer a health benefit to the host (FAO/WHO, 2006). They are active viable bacteria isolated from their natural environment (e.g., gut, reproductive system) in the human body and protect the host after intake. *Lactobacillus* and *Bifidobacteria* are the most common bacterial species used for vaginal probiotics and they can be administered orally or vaginally (Cribby et al., 2008; Fijan, 2014; Buggio et al., 2019; Chee et al., 2020). The theory behind the oral usage of probiotics in the treatment of gynecological disorders is associated with their ability to thrive through the gastrointestinal system to the vaginal tract after their excretion from the rectum. Whilst vaginal administration of probiotics is based on the fact that it allows a direct and targeted colonization action for restoring the unhealthy vaginal microbiota (Homayouni et al., 2014). Precisely in obstetrics and gynecology, lactobacilli strains are mainly used to restore the physiologic vaginal microbiota in order to treat vaginal infections like VVC and also prevent preterm birth (Buggio et al., 2019). This is attributed to their ability in producing lactic acid which gave them a competitive advantage over other microorganisms in the polymicrobial environment. In general, known *Lactobacillus* species including *L. reuteri*, *L. crispatus*, *L. rhamnosus*, *L. acidophilus*, *L. delbrueckii*, and *L. gasseri* are native inhabitants of the female reproductive tract. They often defend the host against fungal colonization through mechanisms such as the secretion of metabolites to inhibit fungal adherence, growth, proliferation, and hypha to biofilm formation (Fuochi et al., 2019; Wegh et al., 2019; Kalia et al., 2020). In essence, LAB strains possess antimicrobial properties that ensures their superiority over other microbes in the microbiota. For instance, EPS produced by LAB strains exhibit antibiofilm abilities (Donot et al., 2012; Matsubara et al., 2016; Angelin and Kavitha, 2020). LAB strains are also able to autoaggregate and coaggregate to enhance probiotic bacteria adherence on the vaginal mucosa surfaces, and subsequently assist in the formation of biofilm-like barrier that prevents pathogen colonization (Malik et al., 2013; Mohanty et al., 2019). For example, in 2002, a study by Ocaña and team provided evidence of autoaggregation between four vaginal lactobacilli strains, that is, *L. salivarius*, *L. gasseri*, and *L. delbrueckii* isolates. Their results also showed co-aggregation of *L. acidophilus* and *L. salivarius* isolates with *Candida* species isolated from the vagina of women with candidiasis (Ocaña and Nader-Macías, 2002). Another *in vitro* study conducted by Jørgensen et al. (2017) reported coaggregation activity between the two *L. reuteri* isolates used in their studies with *C. albicans* strains which proved hostile to the growth of this yeast (Jørgensen et al., 2017). Also, a study by Chew and colleagues identifies the coaggregation of *L. rhamnosus* GR-1 and *L. reuteri* RC-14 to possess anti*candidal* and antibiofilm effects against *C. glabrata* in causing vulvovaginal candidiasis *in vitro*. Confocal laser scanning microscopy also showed antibiofilm activity on the morphology of *C. glabrata* after co-culturing with the *Lactobacillus* strains (Chew et al., 2015). Moreover, coaggregation of *L. fermentum* LF10 and *L. acidophilus* La02 from vaginal swabs of healthy women decreased RVVC by approximately 72% through fungal biofilm inhibition *in vitro* (Murina et al., 2014). Clinically, coaggregation between *Lactobacillus* strains isolated from vaginal flora of healthy women have been reported to induce vaginal colonization of lactobacilli and decreased RVVC in women initially diagnosed with VVC (Anukam et al., 2009; Ehrström et al., 2010). These interactions also describe the positive medical importance of bacterial-fungal relationship between *Lactobacillus* species and *Candida* species in reducing vaginitis through co-aggregation. Antimicrobial peptides (AMPs) such as bovine lactoferrin (BLF) produced by LAB strains have been identified as one of the first line barriers in the host defense against a wide range of pathogens by exerting bactericidal and fungicidal activities against them on the mucosal surfaces (Magana et al., 2020). Due to the above reason, AMPs are being investigated for their potential use in treatment regimen for fungal infections such as VVC (Fernandes and Carter, 2017). As a result, Liao et al. (2019) established a BLF-producing *L. casei* pPG612.1-BLF and evaluated its preventative and therapeutic activity in the murine model of VVC (Liao et al., 2019). Results reported indicated the effective synergism of *L. casei*/pPG612.1-BLF strain treatment in the diseased VVC mice models. This indicated a promising preventative and therapeutic anti-VVC agent by using *L. casei* as a vehicle for biotherapy in the female genital tract and exploiting the natural antibiotic antimicrobial peptides for other applications (Liao et al., 2019). In a recent study conducted by Hefzy and co in 2021, AMPs known as bacteriocin-like substances isolated from probiotic lactobacilli strains exhibited antibiofilm and anticandidal activities against clinically isolated *Candida* species, *in vitro* and *in vivo* (Hefzy et al., 2021). These *Lactobacillus* strains including *L. pentosus*, *L. paracasei* subsp. *paracasei* II, *L. rhamnosus* I, *L. delbrueckii* subsp. *lactis* I, and *S. uberis* II significantly inhibited the growth and biofilms of *C. albicans* and NACS clinical isolates from women with VVC. The probiotic strains were also able to exert antagonism *in vivo* against *Candida* isolates when tested using Galleria mellonella larvae model treated with the *Candida* species (Hefzy et al., 2021).

Only a limited number of clinical trials have been conducted to examine the effect of probiotics for VVC treatment (Williams et al., 2001; Anukam et al., 2009; Martinez et al., 2009). For instance, in the clinical trial by Williams et al. (2001), they evaluated the effectiveness of vaginal administration of probiotics (i.e., *L. acidophilus*) with clotrimazole tablets to treat VVC and its reoccurrence among 164 HIV-infected women, in a randomized, double-blind, placebo-controlled trial. Authors reported a reduction in the recurrence of VVC in their 21-month study in groups treated with both probiotics and clotrimazole as compared to the placebo (Williams et al., 2001). Martinez et al. (2009), also investigated the effects of probiotic capsules containing *L. reuteri* RC-14 and *L. rhamnosus* GR-1 on participants already diagnosed with VVC. These participants took FLZ in addition with either the probiotic capsule or a placebo. At the end of a 4-week period, it was found that those who had been taking the probiotic with the antifungal treatment, experienced a significant improvement in symptoms (e.g., less vaginal discharge) and fewer yeast cells were present in their culture samples compared to those in the placebo group (Martinez et al., 2009). A similar study was undertaken by Anukam et al. (2009) to also evaluate the significance of combining probiotics with antifungals in VVC treatment. The study recruited 59 premenopausal women who had been diagnosed with VVC. The women were advised to take FLZ with either a probiotic capsule containing *L. reuteri* RC-14 and *L. rhamnosus* GR-1, or a placebo. It was found that, significantly fewer of those in the probiotic intervention group had a reoccurrence of their thrush symptoms as compared to diagnosed women who took the placebo (Anukam et al., 2009). In 2010, Ehrström and fellow researchers also conducted a clinical study to assess vaginal colonization of LAB and its clinical outcome in the treatment of VVC, which was undertaken within a 1-year period. The study population consisted of 95 women, of which 45 women had VVC. Participants in the study were administered vaginal capsules containing *L gasseri* LN40, *L. fermentum* LN99, *L. casei subsp. rhamnosus* LN113 and *P. acidilactici* LN23, or placebos vaginally for 5 days after conventional treatment. A total of 399 vaginal samples were sampled and analyzed for the presence of the probiotic strains after the 5 days. Results indicated that all the women were colonized by one or two of the probiotic strains used in the study. The probiotic supplementation in their study also resulted in significantly higher overall clinical cure rate with lesser symptoms as compared to the placebo. In addition, the short treatment period to establishing this cure rate showed that it is very promising. The study encouraged further randomized clinical studies with extended and/or repetitive treatments with probiotic strains, in combination with conventional treatment (Ehrström et al., 2010). Overall, this indicates that conventional antifungal drugs with probiotics, as opposed to being used alone, could enhance the antifungal effect in improving the rate of short-term clinical cure (within 5–10 days), short-term mycological cure and possibly prevent a relapse of the infection (Xie et al., 2017; Jeng et al., 2020).

Despite the fact that probiotics have not been widely prescribed for VVC/RVVC treatments, it can be one potential approach to reduce risk of infections through vaginal dysbiosis by the maintenance of vaginal eubiosis (Barrientos-Durán et al., 2020). Unfortunately, probiotics have not been widely commercialized in the treatment of VVC/RVVC because of the strain-dependency of the efficacy of strains and no confirmed appropriate dosage and duration for use. Also, the exact mechanisms of probiotics are unclear and still been investigated since it exhibits varying efficacies based on targeted groups or individuals (e.g., pregnant, and non-pregnant women), geography, race, and ethnicity (e.g., Caucasian, Asian, Black and Hispanics, White women). For instance, the incidence of vaginal communities in which lactobacilli are not dominant is higher in black women as compared to Caucasian, White and Japanese women in studies (Zhou et al., 2010; Ravel et al., 2011; Borgdorff et al., 2017). The difference and diversity in the composition of the vaginal microbiota of women in these different racial groups, may account for difference in susceptibility to vaginal infections and response to various treatments as well. Furthermore, other factors such as host genetics, environment, traditions and life-style may affect the vaginal composition (Gupta et al., 2017) and probably cause alterations in the role of probiotics strains in different women. This is because, the microbial communities in the host maybe shaped by these factors. As stated by Gupta et al. (2017), there is a need for a global association study between human microbiota and how different geographic locations, diet, ancestry, and locations can influence the human microbiome architecture (Gupta et al., 2017).

### Postbiotics

Postbiotics is a broad term to describe constituents (bioactive compounds) and metabolites secreted by microbial cells during fermentation processes from probiotic microorganisms with equivalent effectiveness as the probiotics (Wegh et al., 2019). The definition of postbiotics have been used interchangeably with that of parabiotics since both of them involve the use of non-viable or cell fractions to confer a health benefit to the consumer when administered in sufficient amounts (Aguilar-Toalá et al., 2018). These non-viable compounds confer beneficial therapeutic outcome to the host without the risk of administering live microbes to impaired immune hosts (Patel and Denning, 2013; Pandey et al., 2015; Compare et al., 2017). This was also reported by other studies that suggested the use of postbiotics as a better alternative in immunocompromised patients amongst other benefits such as longer shelf-life as compared to probiotics (Rampengan et al., 2010; Deshpande et al., 2018). Postbiotics have been classified as the complex mixture of metabolic products secreted by probiotics in their cell free supernatant (CFS) including enzymes, secreted proteins, short chain fatty acids, vitamins, secreted biosurfactants, amino acids, peptides, and organic acids (Nataraj et al., 2020). These CFS containing biologically active metabolites secreted by bacteria and yeast into the surrounding liquid can be obtained directly from cell cultures and is strain dependent (Żółkiewicz et al., 2020). Postbiotics can generate hostile situations such as alterations in the acidic condition of the vaginal environment thereby affecting pathogenic microorganisms gravely and fighting their infections (Amabebe and Anumba, 2018). Hampering of the social interactions and communication system between pathogens is a tool in controlling their virulence through biofilm formation. Probiotic strains are able to secrete metabolites that inhibit quorum sensing activities known as quorum sensing inhibitors. It has been shown that quorum sensing inhibitors can either disrupt the process of biofilm formation or disperse already formed biofilms (Abraham, 2016). The great news is anti-quorum sensing compounds are said to be neither lethal nor cause drug resistance in hosts. Organic acids (e.g., lactic acid) is an example of such anti-quorum sensing compound because they can reduce environmental pH which generates an unfavorable environment for pathogenic activities (Lopes et al., 2017; Kiymaci et al., 2018). In light of this, postbiotics have gained growing interest in the controlling of yeast growth, infections and maintaining a healthy ecosystem in the host (Żółkiewicz et al., 2020). Currently, postbiotics are being isolated to treat invasive candidiasis and other infections (Rossoni et al., 2020). The CFS of LAB strains have been reported in studies to exert antagonism against VVC causing *Candida* and NACS (Chew et al., 2015; Parolin et al., 2021). In these studies, authors reported the successful inhibition of the growth and biofilm formation of *Candida* species isolated from VVC infected women, using the CFS of vaginal isolated *Lactobacillus* strains *in vitro*. Significantly, Parolin explored the synergism of freeze-dried CFS of *L. crispatus* and postbiotic hyaluronic acid in the treatment of VVC (Parolin et al., 2021). For most probiotics strains especially *Lactobacillus* strains, lactic acid and acetic acid are the main organic acids produced which aid in their potency. The presence of these organic compounds produced by LAB species amongst other microbial excretes have proven in studies to be able to prevent vaginal candidiasis by hindering the growth of both *C. albicans* and *C. glabrata* (Chew et al., 2015; Lourenço et al., 2019). These metabolites also have antibiofilm effect against a range of *Candida* species and have been effectively employed in the treatment of VVC both *in vitro* and *in vivo* (Mota et al., 2015; Rahmani et al., 2020). Further, synergism between these organic compounds with antifungal therapy can increases the efficacy of azoles against *Candida* species (Zangl et al., 2019).

The production of biosurfactants (BS) by LAB strains have also been recognized to have antibiofilm effects on vaginal *Candida* species (Itapary Dos Santos et al., 2019; Liao et al., 2019; Hefzy et al., 2021). BS refer to any active surface compounds that lower the surface tension and has emulsifying properties. They are amphiphilic compounds produced by microorganisms, anchored on the surface, or secreted to the outside, with outstanding surface and emulsifying properties (Santos et al., 2016). BS are produced by LAB strains including vaginal *Lactobacillus* species and have anti-adhesive abilities against pathogens with no exception to *Candida* species (Sarwar et al., 2018; Ghasemi et al., 2020). Overall, BS isolated LAB strains in human urogenital and gastrointestinal tracts, have proven to be highly effective in eliminating pathogenic bacteria and fungi adhering on various surfaces including medical implants (Satpute et al., 2016; Merghni et al., 2017; Yan X. et al., 2019). In fact, BS can significantly decrease the adhesion of pathogens in a dose-dependent manner, which affects the expression of biofilm-related genes and interferes with the microbial social activities in quorum sensing of pathogens (Yan X. et al., 2019). In vaginal health, the antibiofilm effects of BS against *Candida* species have been investigated. De Gregorio and colleagues in 2020, isolated BS from vaginal *L. crispatus* and evaluated its antagonism *in vitro* and *in vivo*. The study indicated the successful inhibition of the adhesion and biofilm formation of six clinically isolated *Candida* species (*C. albicans*, *C. glabrata*, *C. krusei*, and *C. tropicalis*) from VVC infected women. Again, *L. crispatus* BC1 biosurfactant has also been proven in a study to counteract *Candida* species biofilms in causing VVC (De Gregorio et al., 2020; Parolin et al., 2021). Other postbiotics (e.g., bacteriocins) have also been used in the treatment and prevention of VVC (Segun, 2015; Fernandes and Carter, 2017; Zangl et al., 2019). Bacteriocins are heat-stable antimicrobial peptides of proteinaceous nature that have bacteriostatic or bactericidal properties that are harmful to pathogens (Kareem et al., 2014). Bacteriocins such as BLF have been investigated for their potential use in treatment regimen for VVC (Liao et al., 2019). Nevertheless, postbiotics have shown a potential to be able to prevent and treat VVC through the various studies that employed derivatives of probiotic strains against *Candida* species and its biofilm formation. A probe into its commercialization can go a long way to help treat and also prevent reoccurrence of VVC.

### Prebiotics

Substantially, further research into the role of biotics in vaginal health, led to the exploitation of prebiotics. Prebiotics play an essential role to keep the gut microbiota and female reproductive system in check by enhancing the growth and viability of probiotic microorganisms (Wegh et al., 2019; Amabebe et al., 2020). Therefore, measures to maintain or encourage the growth of beneficial species such as LAB strains will provide healthy grounds for LAB colonization to prevent fungal adhesion and growth (d’Enfert et al., 2021). The consumption of prebiotic-rich diet is a mechanism to confer such a role in a host and increase host immunity against infections as well (Davani-Davari et al., 2019). As the name suggests, “pre” -biotics which means “before” comes before probiotics and are defined as substrates that are selectively utilized by already existing host microorganisms to confer health benefits (Gibson et al., 2017). Vyas and Ranganathan (2012), describe the role of prebiotics as been able to change the microbiota composition by inducing the growth of non-pathogenic species, thereby promoting health benefits in the host (Vyas and Ranganathan, 2012). Prebiotics when consumed can travel through the body to the desired sites to confer beneficial effects to the host. They can also improve the gut microbiota and also urogenital tracts (Reid, 2012; Kaur et al., 2021). Mizgier et al. (2020) emphasized in their study that, future studies and reviews should investigate the role of diet and probiotics in changes to vaginal microbiome in order to decipher the mechanisms in vulvovaginal candidiasis infections (Mizgier et al., 2020). Prebiotics have a direct effect on microbial growth as they stimulate the growth of beneficial bacteria and suppress the growth of pathogens (Ohshima et al., 2016). A major prebiotic is lactoferrin which is said to be a major defense protein of the innate immune system. It is an iron-binding protein that promotes the growth of LAB strains and have antifungal, antibacterial and antiviral properties (Kell et al., 2020). Lactoferrin can protect the vagina from infections through hyphae and biofilm inhibition (Fais et al., 2017; Curvelo et al., 2019). The antibiofilm activities of lactoferrin against FLZ resistant *Candida* species has been evaluated *in vitro*. The biofilms formed by clinically isolated *C. albicans* strains were inhibited in a dose dependent manner by lactoferrin after XTT evaluation (Curvelo et al., 2019).

### Synbiotics

**Figure 2:**
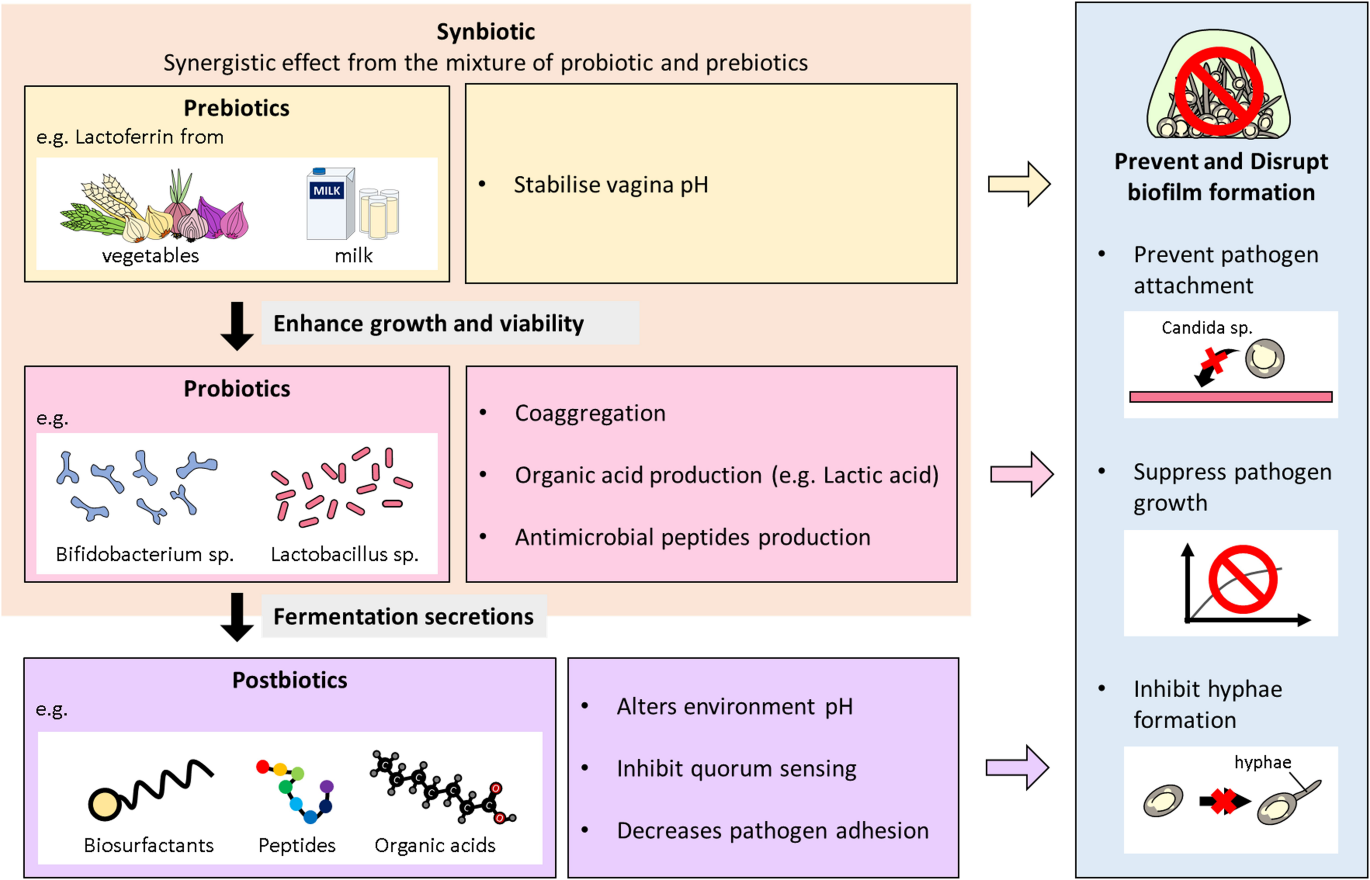
Overview of the antibiofilm role of biotics family in VVC. This diagram illustrates the role probiotics, postbiotics, prebiotics, and synbiotics play in preventing and inhibiting biofilms formation of *Candida* species in VVC infections.

Synbiotics are described as a combination of probiotics and prebiotics, typically oligosaccharides or inulin, which demonstrate an additive action and are used to restore normal bacterial flora (Pandey et al., 2015). Basically, they are the synergism between prebiotics and probiotics to aid probiotics growth and viability in the host (Gurry, 2017). Overall, prebiotics like fructooligosaccharides, galactooligosaccharides, Inulin; fructans are the most commonly used fibers with probiotics and termed as synbiotics (Pandey et al., 2015). The combination instead of the single use of either probiotics or prebiotics, is to give superior outcome than when taken independently. Synbiotics can boost probiotics action by modulating essential metabolites production (Kvakova et al., 2021). These microbial excretes can in turn exert hostility including biofilm inhibition against pathogens. Ideally, studies using synbiotics have also been reported in vaginal infections (Al-Ghazzewi and Tester, 2016; Liao et al., 2019; Superti and De Seta, 2020). The synergistic effects of *L. acidophilus* and *L. rhamnosus* isolates in combination with BLF in treating VVC and RVVC has been evaluated *in vitro* (Superti and De Seta, 2020). Also, the synbiotic effects of probiotic strains (*L. acidophilus* GLA-14/*L. rhamnosus*) and (BLF + clotrimazole) was also assessed in a randomized clinical trial (Russo et al., 2019). The combined regimen was tested on women with RVVC within 3–6 months. A follow-up after 6 months indicated a reduction of the reoccurrence of VVC in the women. Again, Liao et al. (2019) also combined the effects of *L. casei* strain which secreted BLF against vaginal *C. albicans* in BALB/c mice (Liao et al., 2019). The results showed that the synbiotic was able to improve the immunity of the vaginal mucosa against *C. albicans*. A representative diagram of the role Biotics family play in preventing and inhibiting biofilms formation of *Candida* species in VVC infections is illustrated below in Figure 2.

## Synergistic Therapy Against Biofilms Involved in VVC

To curb the role of biofilms in vaginal fungal infections, novel and synergistic regimen are being investigated intensively. Most of these approaches are adjunctives which entails already existing remedies in combination with biotics, antifungals, nanoparticles, and plant extracts to enhance their effectiveness (Genovese et al., 2019; Liu et al., 2020; Melo et al., 2020; Pandey et al., 2020). This synergism will target the adhesion of pathogens, their biofilm formation, cell to cell interactions and also destroy persister cells (Koo et al., 2017). As for the eradication of fungal biofilms in VVC and RVVC, highly recommended remedies include combining biotics with antifungals, drugs, plant extracts and nanoparticles to give synergistic effects (Table 2). This synergism will aid in interrupting quorum sensing of fungal species, weaken and in consequence inhibit biofilms. The synergistic effects of both conventional and non-conventional regimens can go a long way to tackle emergence of drug resistance of biofilm positive microbial species as well (Jiang et al., 2020). Generally, synergism between drugs is very relevant to prevent drug resistance and increase efficacy of the treatment by expanding the drug activity spectrum. Synergism between drugs can prevent emergence of drug resistance by closing the mutant selection window of each other and dispensing different mechanism in the host. For example, synergism between FLZ and amphotericin B, can induce different drug actions and increase treatment effect. A mutant selection window is a concept proposed by Drlica and Zhao (2007), which hypothesizes that, there is a drug concentration zone in which resistant mutants of pathogens are selectively amplified, inducing reduced drugs susceptibility (Drlica and Zhao, 2007). However, his form of synergism can also lead to multidrug resistance because, the frequent usage of OTC drugs in the main also contributes to resistance by pathogens including *Candida* species (Bordallo-Cardona et al., 2018). Therefore, one of the goals in acute infections treatment will be to reduce the unnecessary use of antibiotics and antifungals. This is why it may be a great idea to combine antifungals with other alternative treatment options such as biotics as an antibiofilm strategy in the treatment of VVC. Fortunately, in the year 2015, results from a clinical study by Kovachev and Vatcheva-Dobrevska, 2015 involving 436 women with VVC proved that, combining a prescribed antifungal medication such as FLZ (Diflucan) with probiotic vaginal suppositories, increases the effectiveness of FLZ. The vaginal probiotic contained *L. acidophilus*, *L. rhamnosus*, *Streptococcus thermophilus*, and *L. delbrueckii subsp. bulgaricus* which was administered after azole treatment. This local application of probiotics after administration of combined azoles for treatment of vaginal *C. albicans* infections increased therapy efficacy and can possibly prevent relapse of the infection. Furthermore, the idea of combining antifungals and biotics is to present different pharmacokinetics and pharmacodynamics parameters in the treatment of VVC *in vivo*. Undoubtedly, the reduced access of drugs to targeted pathogens in a biofilm matrix, is a major virulence and resistance mechanism of *Candida* species. However, in the treatment of VVC, antifungals can be fungicidal/fungistatic or both (Odds et al., 2003; Mesa-Arango et al., 2014). Therefore, adaptability mechanisms such as biofilm formation and persister cells formation exploited by fungi may obstruct the functions of antifungals (Bink et al., 2011; Tits et al., 2020; Wu et al., 2020). Fortunately, probiotics and postbiotics specifically have been identified in many *in vitro* and *in vivo* studies, to have quorum sensing inhibition abilities, anti-hyphae, antibiofilm and immunomodulatory properties (Villena et al., 2011; Hardy et al., 2013; Jørgensen et al., 2017; Li et al., 2019; Ribeiro et al., 2020; Vazquez-Munoz and Dongari-Bagtzoglou, 2021). It will be a good idea to counteract pathogens by introducing a combined therapy of azoles with probiotics/postbiotics to disrupt their protective shield against antifungals through biofilm formation. This will also prevent multidrug resistance due to less usage of various antifungals. Specifically, lactic acid which is an autoinducer inhibitor is produced by probiotics. Its combination with antifungals can reduce risk of resistance since it will prevent hyphae and biofilm formation which is an emerging reason for resistance and reoccurrence of VVC in vaginal fungal infections. Additionally, in a prospective comparative study by Abdelmonem et al. (2012), which included 129 pregnant patients with VVC found that, a mixture of honey and yogurt had effects similar to traditional antifungal medications. The yogurt and honey mixture was better at reducing symptoms, while the antifungal medication was more effective for eliminating fungi (Abdelmonem et al., 2012).

**Table 2:** Summary of studies conducted on synergistic therapy of biotics with various regimens against VVC and its reoccurrence.

Synergistic therapy	Synergistic effect	References
Hyaluronic acid + *L. crispatus* (lyophilized CFS)	Anti-*Candida* activity was enhanced against all *Candida* species by (hyaluronic + *L. crispatus* lyophilized CFS) as compared to being tested against *Candida* species individually.	Parolin et al., 2021
Amphotericin B-loaded Eudragit RL100 nanoparticles + hyaluronic acid	Inhibition of *C. albicans* growth by (hyaluronic acid + amphotericin B-loaded nanoparticles) *in vitro* and rapid *C. albicans* elimination in the vagina of rats submitted to the VVC model.	Melo et al., 2020
Acetylsalicylic acid + fluconazole	Antibiofilm activity of (acetylsalicylic acid + fluconazole) reported against all clinical and reference vaginal *C. parapsilosis* strains.	Santos et al., 2020
Tacrolimus (FK506) + fluconazole	Tacrolimus in addition to fluconazole exhibited remarkable resistance reversal in all *C. tropicalis* isolates as compared to being used alone against the strains.	Pandey et al., 2020
*L. reuteri* CRL 1324 + *L. rhamnosus* CRL 1332	Inhibition of *C. albicans* vaginal colonization and inflammatory response in VVC induced BALB/c mice. The preventive or therapeutic schemes of the probiotic strains did not show a protective effect as compared to the two probiotics (*L. reuteri* CRL 1324 + *L. rhamnosus* CRL 1332) combined.	De Gregorio et al., 2019
Tioconazole + chitosan-hydroxypropyl methylcellulose	The formulation of (tioconazole + chitosan-hydroxypropyl methylcellulose) exhibited faster antimicrobial activity against vaginal *C. albicans* isolates.	Calvo et al., 2019
(Fluconazole/metronidazole/clotrimazole) + oral probiotic	Synergistic effect observed between combined antifungals and oral probiotic used as an adjuvant in treatment of RVVC.	Genovese et al., 2019
*L. casei* + bovine lactoferrin (pPG612.1-BLF)	Inhibition of *C. albicans* growth by (*L.casei* + blf) and reduction in fungal burden of VVC induced mice after 5 days treatment.	Liao et al., 2019
*L. acidophilus* GLA-14/*L. rhamnosus* and bovine lactoferrin + clotrimazole	Synergistic activity recorded in women treated with the combined therapy within 3–6 months. Follow-up after 6 months showed reduction of reoccurrence of VVC in reported cases due to this combination.	Russo et al., 2019
Lactoferrin + fluconazole	Synergistic activity of (LF + fluconazole) against fluconazole-resistant vaginal *C. albicans* strains.	Fernandes and Carter, 2017
Probiotics + antifungal drugs (azoles)	Synergistic enhanced activity of (probiotics + azoles) within 5–10 days compared to conventional antifungal drugs used individually.	Xie et al., 2017
Glycyrrhetinic acid + hyaluronic acid	Synergistic antifungal activity against *C. albicans* and non*-Candida* species isolated from RVVC patients.	Stevan et al., 2017
*L. plantarum* I1001 + clotrimazole	Synergistic activity of *L. plantarum* isolate + clotrimazole at preventing recurrence of VVC.	Palacios et al., 2016
Quercetin + fluconazole	Synergistic antifungal activity of (quercetin + fluconazole) in the clinical management of VVC caused by *C. albicans. In vivo*, fungal burden was reduced in vaginal mucosa and symptoms were alleviated after treatment.	Gao et al., 2016
*L. reuteri RC-14 and L. rhamnosus GR-1* + fluconazole	Synergistic activity of (*L. reuteri* RC-14 and *L. rhamnosus* GR-1 + fluconazole) against yeast *in vitro* and up-regulation of anti-inflammatory cytokines IL-8 and IP-10 by human vaginal cells (VK2/E6E7). At 4 weeks, (*L. reuteri* RC-14 and *L. rhamnosus* GR-1 + fluconazole) treated group showed significantly less vaginal discharge associated with symptoms and lower presence of yeast detected by culture.	Martinez et al., 2009

On that account, Table 2 was constructed to highlight the importance of the possibility of the synergism of antifungals with pro-, pre-, post-, and synbiotics. In consequence to the increasing rise of antifungal resistance in association with fungal biofilm formation, it is highly recommended in this study to combine drugs with anti-biofilms agents (e.g., probiotics, postbiotics), coupled with a good diet plan (prebiotics) to diminish this predicament and reduce invasiveness of pathogens. Therefore, Table 2 is a summary of recently reported studies that combined drugs with biotics, to give synergistic effects against biofilms formed by *Candida* species isolated from VVC/RVVC patients.

## Conclusion

Vaginal candidiasis has been identified as an opportunistic vaginal mucosal infection and the second most common cause of vaginitis in women after BV. This infection accounts for about 7% of gynecological visits and characterized by a thick white discharge coating the walls of the vagina, usually accompanied by no odor as opposed to BV which has an unpleasant fishy odor. *Candida* species establish infections by adhering to vaginal surfaces and forming biofilms which further leads to infections. Therefore, understanding biofilm formation of *Candida* species, their structure, development, morphology, and the role they play in causing VVC is a vital step in eradicating this fungal invasion. It is well documented that *Candida* species exert their cohesion and tolerance to antifungals and host immune system through this mechanism. Therefore, novel options with synergistic abilities to not only prevent fungal growth, adhesion and biofilm development but also inhibit them eventually is highly proposed in many studies. In this review, we conclude and recommend the combination of already existing antifungals together with the biotics family due to their potent antibiofilm and antifungal activities. Specifically, besides the better-known probiotics, equal emphasis ought to be placed on utilizing postbiotics from vaginal LAB due to the significant antibiofilm effects of the metabolites produced. As in many other approaches in tackling diseases, the age-old advice of “Prevention is better than cure” still holds true. In addition to searching for treatment, it is also necessary to educate women on the importance of a healthy lifestyle in terms of maintaining proper hygiene, avoid overusing or not using at all feminine products and a proper diet for sustaining a healthy vaginal microflora.

## Author Contributions

AB conceptualized, designed, and wrote the review. SC and LT reviewed and edited the manuscript. AB and Y-LL designed and illustrated the figures in the manuscript. All authors contributed to the article and approved the submitted version.

## Conflict of Interest

The authors declare that the research was conducted in the absence of any commercial or financial relationships that could be construed as a potential conflict of interest.

## Publisher’s Note

All claims expressed in this article are solely those of the authors and do not necessarily represent those of their affiliated organizations, or those of the publisher, the editors and the reviewers. Any product that may be evaluated in this article, or claim that may be made by its manufacturer, is not guaranteed or endorsed by the publisher.

## Abbreviations

AIDS: Acquired immunodeficiency syndrome
*ALS*: Agglutinin-like sequence
AV: Aerobic vaginitis
BLF: Bovine lactoferrin
BS: Biosurfactants
BV: Bacterial vaginosis
CFS: Cell free supernatant
CST: Community state types
ECM: Extracellular matrix
*EPA3*: Epithelial adhesin 3
EPS: Extracellular polymeric substances
*ERG11*: Lanosterol 14-alpha-demethylase
FLZ: Fluconazole
*HWP*: Hyphae wall protein
IUDs: Intrauterine devices
LAB: Lactic acid bacteria
NACS: Non-*albicans Candida* species
OTC: Over-the-counter
*PIR1*: Cell wall mannoprotein
QSMs: Quorum sensing molecules
RVVC: Recurrent vulvovaginal candidiasis
TV: Trichomonas vaginitis
VVC: Vulvovaginal candidiasis
